# A Smart System for an Assessment of the Remaining Useful Life of Ball Bearings by Applying Chaos-Based Health Indicators and a Self-Selective Regression Model

**DOI:** 10.3390/s23031267

**Published:** 2023-01-22

**Authors:** Shih-Yu Li, Hao-An Li, Lap-Mou Tam, Chin-Sheng Chen

**Affiliations:** 1 Graduate Institute of Manufacturing Technology, National Taipei University of Technology, Taipei 10608, Taiwan; 2 Institute for the Development and Quality, Macau, Macao 999078, China; 3 Department of Electromechanical Engineering, Faculty of Science and Technology, University of Macau, Macao 999078, China; 4 Graduate Institute of Automation Technology, National Taipei University of Technology, Taipei 10608, Taiwan

**Keywords:** smart assessment, remaining useful life (RUL), chaos-based health indicators (CHI), regression analysis, iterative cumulative moving average (ICMA)

## Abstract

Bearings are the most commonly used components in rotating machines and the ability to diagnose their faults and predict their remaining useful life (RUL) is critical for system maintenance. This paper proposes a smart system combined with a regression model to predict the RUL of bearings. The method converts the azimuth signal through low-pass filtering (LPF) and a chaotic mapping system, and uses Euclidean feature values (EFVs) to extract features in order to construct useful health indicators (HIs). In fault detection, the iterative cumulative moving average (ICMA) is used to smooth the HIs, and the Euclidean norm is used to find the time-to-start prediction (TSP). In terms of prediction, this paper uses a self-selective regression model to select the most suitable regression model to predict the RUL of the bearing. The dataset provided by the Center for Intelligent Maintenance Systems (IMS) is applied for performance evaluation; in comparison with previous research, better prediction results can be achieved by applying the proposed smart assessment system. The proposed system is also applied to the PRONOSTIA (also called FEMTO-ST) bearing dataset in this paper, demonstrating that acceptable prediction performance can be obtained.

## 1. Introduction

The real-time monitoring of ball bearings is a core point issue in the current age of developed technology—not only to facilitate the arrangement of maintenance schedules but also to avoid missing faults in the ball bearings with different fatigue levels. As modern industries inevitably utilize a wide range of rotating machinery that uses ball bearings, the need to ensure safety during their service life has increased significantly. As time goes on, mechanical systems increasingly rely on prognostic and health management (PHM) [1,2,3] to maintain the safety and maintenance of the entire production line.

The PHM system can be divided into three stages: construction of system health indicators (HIs), prediction of the remaining useful life (RUL) of the system, and health management (HM) [3]. In the construction of HI values, there are a number of technical indicators to present the health status of the system, such as the root mean square (RMS) [4], kurtosis [5], entropy [6], and Mahalanobis distance (MD) [7]. In RUL prediction, the current state of health indicators is detected. When the health indicators are abnormal, the system determines the failure and starts to predict the remaining service life. The predicted time is used to schedule system-related maintenance measures. Ball bearings are the most common mechanical components in mechanical systems, and their health status and RUL prediction have attracted the attention of many scholars [8,9,10,11,12,13,14,15,16]. When a bearing fails, it can lead to increased power consumption and the shutdown of engineered systems which, in turn, affects manufacturing costs. Therefore, being able to predict the fault location in advance and estimate the RUL of the bearing is crucial for the maintenance of system components and avoiding the sudden stoppage of the system.

The research on bearing health and RUL prediction can be mainly divided into model-based [8,9,10] and data-driven [11,12,13,14,15,16,17] research. Model-based methods refer to the setup of a mathematical or physical model describing the degradation process of a mechanical system. For example, Gebraeel et al. [8] used a Bayesian approach to monitor bearing information in real-time in order to update exponential model parameters and estimate bearing life. Li et al. [9] proposed an improved exponential model of bearing prediction, using a particle filter to remove random errors in the exponential model. In [10], the authors used a weighted minimum quantization error to construct HIs and performed RUL prediction using a maximum likelihood estimation algorithm and a particle-filter-based algorithm. However, although these methods have good predictive results, model-based methods also have limitations. To be applied to other systems based on the model, it is necessary to remake a specific model, increasing the development cost.

Data-driven methods refer to using machine learning to train the run-to-failure process of a mechanical system and extract key features of degradation to construct an RUL prediction system. For example, Loutas et al. [11] used wavelet analysis to extract bearing features and used support-vector regression (SVR) to predict bearings’ RUL. Tran et al. [12] used residual-based root mean square and Cox proportional hazards models and support-vector machines (SVMs) for RUL prediction. The authors of [13,14] both used deep learning to predict the RUL of bearings. Guo et al. [15] proposed a method based on a recurrent neural network (RNN) to extract 14 kinds of bearing features to construct HIs. Caesarendra et al. [16] used three models—a relevance vector machine (RVM), logistic regression (LR), and autoregressive moving average/generalized autoregressive conditional heteroscedasticity (ARMA/GARCH)—to evaluate shaft bearings’ degradation. Wang et al. [17] detected bearing degradation through signal deviation, and the bearings’ RUL prediction was performed using an enhanced Kalman filter and an expectation maximization algorithm. Recently, many researchers have proposed hybrid methods. Ahmad et al. [18] used adaptive regression to select the best regression model to predict bearings’ RUL. In [19], an exponential weighted moving average (EWMA) control chart combining SVR and random forest regression (RFR) with a differential evolution (DE) algorithm was proposed to predict ball bearings’ RUL. In [20], Cho proposed a gated recurrent unit (GRU) that can better handle large training data. The GRU synthesizes a single update gate with the forgetting gate and the input gate. Meanwhile, in [21], a local-feature-based GRU was applied to verify the effectiveness of machine health monitoring tasks. Moreover, in [22,23], the relevance vector machine (RVM) method was applied to further predict the RUL of a gear system under progressive wear (i.e., fatigue pitting).

In the context of these previous works, this paper proposes a hybrid technique for the prediction of bearings’ RUL, using a low-pass filter (LPF) to efficiently extract specific frequencies and applying a chaotic mapping strategy [20] combined with Euclidean feature values (EFVs) [21] to construct chaotic Euclidean feature values (CEFVs) as the bearings’ health indicators (HIs), which have a positive correlation with the bearings’ degradation, where the iterative cumulative moving average (ICMA) is adopted to smooth the HIs. Additionally, we select the most suitable regression model to predict RUL according to the development trend of the CEFVs, which are also confirmed to be effectively applicable to fault diagnosis [24,25,26].

The remainder of this paper is organized as follows: In Section 2, the complete process for the RUL prediction of ball bearing systems is presented step-by-step. In Section 3, the experimental results are presented and compared with other methods. Finally, a conclusion is provided in Section 4.

## 2. Materials and Methods

**Figure 1:**
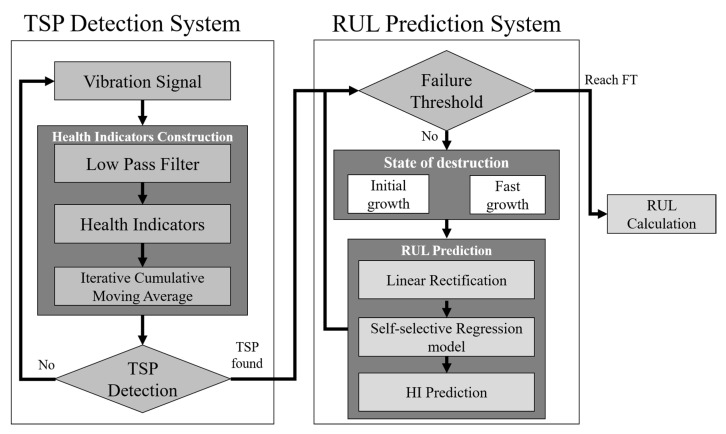
The proposed bearing RUL prediction system.

The system presented in this paper can be divided into two parts, as shown in Figure 1; the first part is the TSP detection stage, which aims to detect the time-to-start prediction (TSP) of degeneration, while the second stage is the assessment of remaining useful life (RUL). The collected mechanical signals of the bearing are extracted to a specific frequency through LPF and ICMA to eliminate noise and singular values, and the HI value is established to detect whether the bearing has degraded. When the time-to-start prediction (TSP) [27] is detected, it will enter the RUL estimation system and extract the latest bearing data in order to build a regression model to predict the RUL of the bearing.

### 2.1. Data Experiment Platform

This paper uses two experimental databases: the first is the Center for Intelligent Maintenance Systems (IMS) [24] database, used to construct our method, while the second is the PRONOSTIA (also called FEMTO) bearing dataset [28], used for validation.

#### 2.1.1. IMS Database

This database consists of experimental data collected by the NSF I/UCR Center for Intelligent Maintenance Systems [29] for rolling bearings’ run-to-failure testing which are available from the public database of NASA Ames Prognostics [30]. The experimental data acquisition platform is also presented in [24], where four bearings were installed on one shaft for simultaneous detection.

The experiment was performed three times, as shown in Table 1. The conditions of the three experiments were all the same. The rotation speed was 2000 rpm, the load was 6000 lb, the sampling rate was 20 kHz, and the vibration signal of 1 s was captured every 10 min through the NI DAQ card 6062E (except for the first 43 files of Test 1 every 5 min) [30]. Each acquisition resulted in a separate file of 20,480 points. The experimental conditions and results are shown in Table 1. In Test 1, Bearing 4 had a ball fault and Bearing 3 had a slight inner-ring fault. In Test 2, Bearing 1 had an outer-ring fault. In Test 3, Bearing 3 had an outer-ring fault.

#### 2.1.2. The PRONOSTIA (Also Called FEMTO-ST) Bearing Dataset

The PRONOSTIA (also called FEMTO-ST) bearing dataset [29] is an experimental platform dedicated to testing and validating methods for the detection, diagnosis, and prediction of bearing faults. The platform was designed and implemented by the AS2M department of the FEMTO-ST Institute, and the experimental platform is described in [29].

For the PRONOSTIA dataset, data were generated by applying the maximum load of the bearing, to accelerate the degradation, using three different load and speed tests. In order to ensure the safety of the platform, the test was stopped when the vibration signal amplitude exceeded 20 g [31]. The experimental conditions are summarized in Table 2. In each experiment, two strokes were selected as training data, and the rest were used as test data. The first case used a radial load of 4000 N and a rotational speed of 1800 rpm; the second case used a radial load of 4200 N and a rotational speed of 1650 rpm; and the third case used a radial load of 5000 N and a rotational speed of 1500 rpm. The sampling rate was 25.6 kHz, and 2560 samples were recorded every 10 s (i.e., 1/10 of a second was collected every 10 s). The vibration signal was captured in the horizontal and vertical directions; we used the horizontal signal for our experiments.

### 2.2. Construction of Health Indicators

The HI construction of this paper can be divided into three parts: the low-pass filter (LPF), chaotic Euclidean feature values (CEFVs), and iterative cumulative moving average (ICMA), which are introduced one by one.

#### 2.2.1. Low-Pass Filter

In the process of collecting the bearing data, external factors may cause noise. Therefore, in this experiment, an LPF was used to filter the noise first. The experiment used the LPF pair of each bearing datum to filter the 3000 Hz response frequency after several tests.

#### 2.2.2. Chaotic Euclidean Feature Values

**Figure 2:**
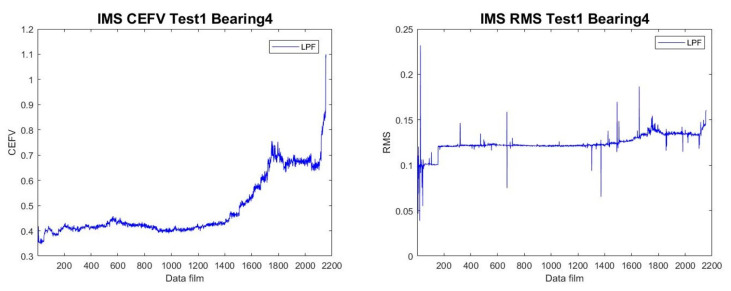
Comparison of CEFV and RMS HI trends.

After the data pass through the LPF, the HI value can be constructed. The HI converts the signal through feature extraction to display the trend graph of the bearing’s degradation status. We used CEFVs to extract HI values from the data because CEFVs can adequately represent the process of bearing degradation. Compared with the RMS value commonly used in the past, CEFVs can amplify the value and can better eliminate the unstable signals in the value, as shown in Figure 2.

The CEFV is a new HI value that combines the chaotic mapping system and the Euclidean feature value (EFV) [32]. The chaotic mapping systems used in this study were two identical Chen–Li chaotic systems [33]—one as the main system and the other as the data feeding system [32], as shown in Equations (1) and (2), respectively.

(1) $$\left\{\begin{array}{l} \dot{x_{1}}=-x_{2}x_{3}+ax_{1}\\ \dot{x_{2}}=x_{1}x_{3}+bx_{2}\\ \dot{x_{3}}=x_{1}x_{2}/3+cx_{3} \end{array}\right.$$

(2) $$\left\{\begin{array}{l} \dot{y_{1}}=-y_{2}y_{3}+ay_{1}\\ \dot{y_{2}}=y_{1}y_{3}+by_{2}\\ \dot{y_{3}}=y_{1}y_{2}/3+cy_{3} \end{array}\right.$$

The chaotic dynamic error state was set to *e*(t) = [$e_{1}\left(t\right)$,$e_{2}\left(t\right)$,$e_{3}\left(t\right)$], where $e_{1}$ = $x_{1}-y_{1}$, $e_{2}$ = $x_{2}-y_{2}$, $e_{3}$ = $x_{3}-y_{3}$; the chaotic mapping system obtained is shown in Equation (3):

(3) $$\left\{\begin{array}{c} {\dot{e}}_{1}={\dot{x}}_{1}-{\dot{y}}_{1}\\ {\dot{e}}_{2}={\dot{x}}_{2}-{\dot{y}}_{2}\\ {\dot{e}}_{3}={\dot{x}}_{3}-{\dot{y}}_{3} \end{array}\right.$$

**Figure 3:**
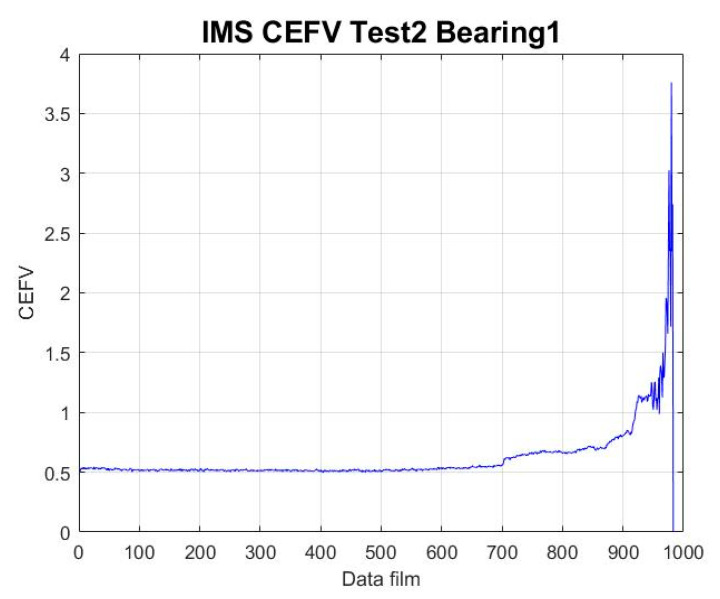
IMS data’s CEFV HI values.

We inserted the IMS Test 2 Bearing 1 data from this experiment into the chaotic mapping system and calculated the dynamic error. Next, we calculated the Euclidean distance (ED) of the obtained dynamic error and extracted the EFV through Equation (4), and then we repeated the execution to convert the experimental data of IMS Test 2 Bearing 1 into CEFVs. The results are shown in Figure 3.

(4) $$\mathrm{EFV}=\frac{\sum_{i=1}^{n}ED_{i}}{n},\;ED=\sqrt{{\left(x_{c}-x_{i}\right)}^{2}+{\left(y_{c}-y_{i}\right)}^{2}+{\left(z_{c}-z_{i}\right)}^{2}}$$

#### 2.2.3. Iterative Cumulative Moving Average

**Figure 4:**
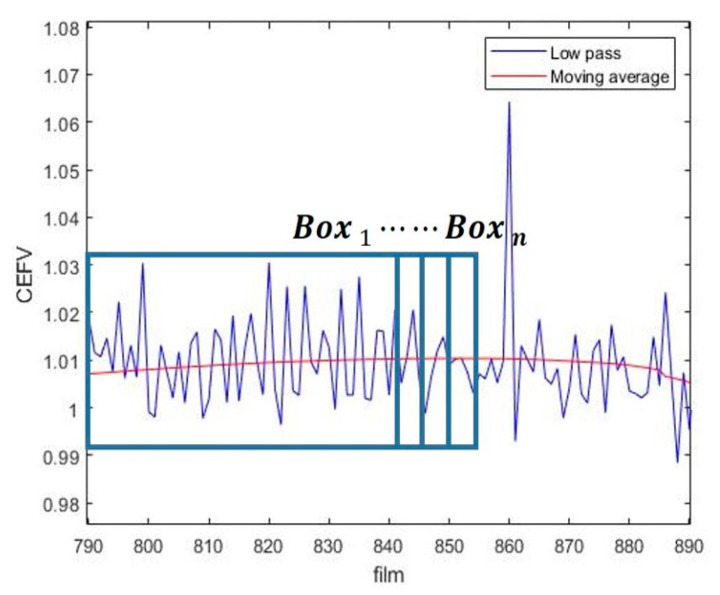
ICMA evolution.

In this study, the iterative cumulative moving average (ICMA) was used to smooth the bearing data in order to improve the overall prediction results. The schematic is shown in Figure 4. When $Box_{1}$ is selected and goes through a simple moving average (SMA), $Box_{1}$ moves forward by one grid, and another SMA is performed for the updated $Box_{2}$ until $Box_{n}$ reaches the maximum data length, as shown in Equation (5), where ${\bar{P}}_{box}$ represents the value after the SMA:

(5) $$ICMA=\frac{{\bar{P}}_{box}+{\bar{P}}_{box-1}+\cdots +{\bar{P}}_{box-\left(m-1\right)}}{m}$$

**Figure 5:**
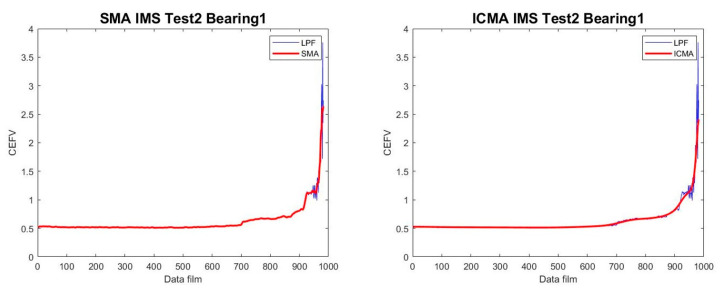
Comparison of the SMA and ICMA results.

The smoothness of ICMA was better than that of SMA when the m value was set to 7. The comparison chart is shown in Figure 5.

### 2.3. Time-to-Start Prediction

**Figure 6:**
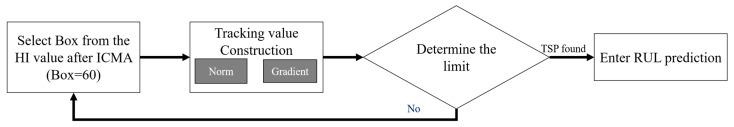
TSP detection process.

Time-to-start prediction (TSP) [27] indicates the first time a bearing begins to fail. The TSP detection process of this experiment is shown in Figure 6. First, we selected a window box to establish tracking for the HI values. When the constructed tracking value exceeds a certain limit, it indicates that the bearing has started to fail, and the RUL is predicted. Two methods were used for constructing the tracking values in this paper: the Euclidean norm, and the gradient, as introduced in Section 2.3.1 and Section 2.3.2, respectively.

#### 2.3.1. Euclidean Norm

The Euclidean norm (norm) refers to the Euclidean distance of a vector in the Euclidean space, which is calculated as shown in Equation (6):

(6) $$norm=\sqrt{x_{1}{}^{2}+x_{2}{}^{2}+\ldots +x_{n}{}^{2}}$$

The first step converts the selected data (window box = 60) to the Euclidean norm; the Euclidean distance of the selected data is calculated, and the norm tracking value $Norm_{i}$ is established to track the current state of the bearing. When $Norm_{i}$ exceeds a certain value, the TSP is detected; the detection method is shown in Equation (7). When the current tracking value $Norm_{i}$ is greater than $M\times Norm_{mean}$, the point is determined to be a TSP. $Norm_{mean}$ represents the average of all established tracking values, including the current one.

(7) $$\left\{\begin{array}{l} Norm_{i}\leq \;M\times Norm_{mean}\;,\;Continue\;detection\\ Norm_{i}>M\times Norm_{mean}\;,\;TSP\;\mathrm{obtained} \end{array}\right.$$

#### 2.3.2. Gradient

The gradient method [26] entails fitting a linear regression model to the window box data, as shown in Equation (8). The regression parameters $\omega_{g}$ and γ are calculated by the least squares method, and $\omega_{g}$ is the required gradient. The gradient detection condition sets a limit value; when the gradient exceeds this limit value, the TSP will be detected.

(8) $$y=\omega_{g}x+\gamma$$

### 2.4. Failure Threshold

The failure threshold (FT) is the stopping point of the predicted RUL, and it also represents the endpoint of the bearing’s life. When the FT is reached, the prediction of the new HI value will be stopped, and the RUL of the bearing will be calculated. In this study, the FT continues to use the norm and gradient tracking values of the TSP, and the FT of the norm value in this research uses $Norm_{TSP}$, where $Norm_{TSP}$ is the $Norm_{mean}$ when the TSP is detected. When the predicted data are converted into tracking values after $Norm_{i}$ exceeds a certain limit, the bearing is identified to fail. When detection does not reach the FT, the detection of fast growth [34] is carried out, which means that the bearing’s degradation will expand, and the HI trend will also increase rapidly.

### 2.5. RUL Prediction

#### 2.5.1. Linear Rectification

In order to prevent the captured data from being too flat, causing the predicted values of the regression predictions to develop into negative values, we used linear rectification (LR) to adjust the curve of the data in the prediction window box in order to improve the accuracy of the regression model and the predicted RUL. The adjustment method can be divided into growth rate adjustment and HI value adjustment.

**Figure 7:**
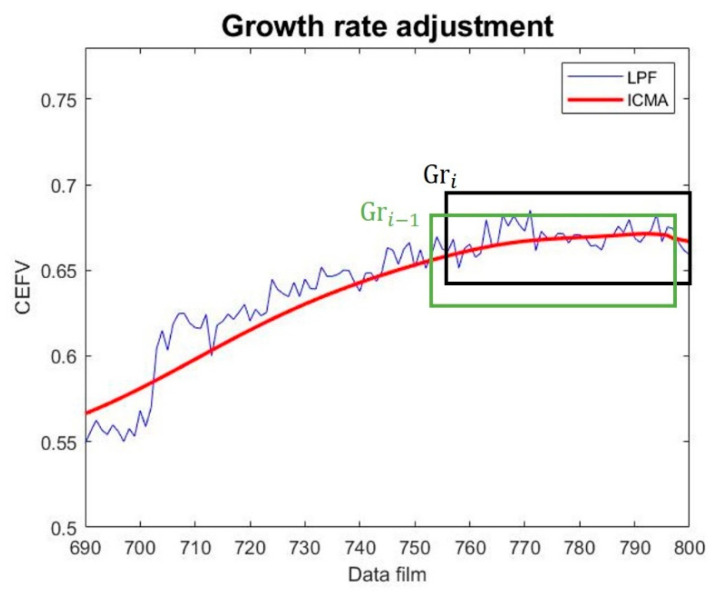
Growth rate adjustment diagram.

Figure 7 shows the growth rate adjustment, and the growth rate is calculated as shown in Equation (9). When the current window box growth rate ${\;\mathrm{Gr}}_{i}$ is less than the previous growth rate ${\;\mathrm{Gr}}_{i-1}$, the growth rate is adjusted. The growth rate adjustment is calculated as shown in Equation (10).

(9) $$\mathrm{Gr}=\frac{1}{box-1}\overset{box}{\underset{n=2}{\sum }}y_{n}-y_{n-1}$$

(10) $$\left\{\begin{array}{c} Gr_{i}=Gr_{i-1}\;,\;\;Gr_{i}<Gr_{i-1}\\ Gr_{i\;},\;\;\;\;\;\;\;\;Gr_{i}\geq Gr_{i-1}\; \end{array}\right.\;$$

**Figure 8:**
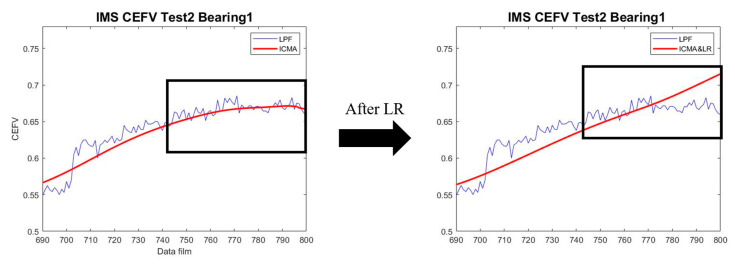
The HI results after LR.

In the HI value adjustment, when the Gr is adjusted, the window box data are adjusted. The HI value adjustment is calculated as shown in Equation (11). The window box data are adjusted from the second datum in the selected data until the last. As shown in Figure 8, the HI growth trend can develop upward after LR.

(11) $$\left\{\begin{array}{c} y_{j}=y_{j-1}+Gr\;,\;y_{j}<y_{j-1}\vee \;y_{j}>y_{j-1}+Gr\\ \;y_{j}\;,\;y_{j-1}\leq y_{j}\leq y_{j-1}+Gr \end{array}\right.\;$$

#### 2.5.2. Self-Selective Regression Model

In this study, after determining the generation of the TSP, a window of size n was constructed from the point of failure, and a self-selective regression model was established by using the HI selected in the window. The opening direction established by quadratic regression automatically selects the most suitable polynomial regression model. Equations (12) and (13) are the quadratic regression and self-selective regression model equations, respectively. The constructed model was used to predict the predicted HI values.

(12) $$y=\beta_{1}x^{2}+\beta_{2}x+\gamma$$

(13) $$\left\{\begin{array}{c} y=\beta_{3}x^{2}+\beta_{4}x+\gamma \;,\;\beta_{1}>0\\ y=\beta_{5}x+\gamma \;\;,\;\;\beta_{1}<0 \end{array}\right.\;$$

**Figure 9:**
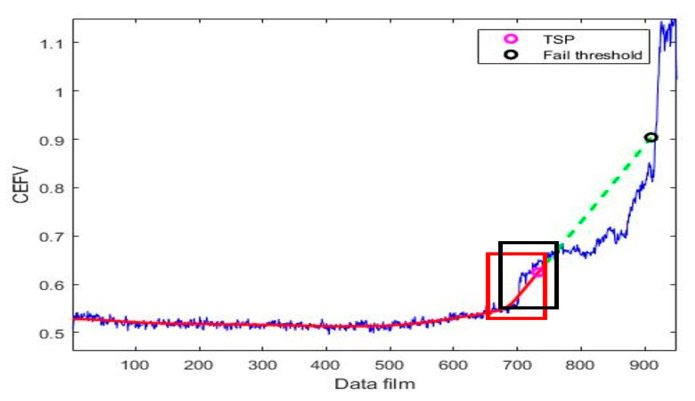
Polynomial regression prediction diagram.

We used the regression prediction proposed in [18] as the basis to make predictions by fitting a self-selective regression model on the nearest n data points ($x_{1}$…$x_{n}$,$\;y_{1}$…$y_{n}$). As shown in Figure 9, we selected the data in the red window box to construct a self-selective regression model to predict the predicted HI values. After the prediction is successful, the window box moves forward by one cell, and the new data points are updated to the new window box. A self-selective regression model was re-established for the data in the window box to predict the next HI value, and the window box moved forward by one grid to update the data; we repeated this step until the predicted result exceeded the FT, at which point we stopped the prediction and started to calculate the RUL.

## 3. Results and Discussion

In this study, the bearings’ RUL was predicted using a self-selective regression model, and the training and testing process was organized as follows: First, the prediction parameters were established on the test data and brought back to the training data for RUL prediction, and the cumulative relative accuracy (CRA) [27] was calculated. By adjusting the setting parameters, we found the most suitable prediction parameters and calculated the CRA. We then substituted the established prediction parameters into the test data to calculate the RUL. In this study, the time for each prediction was ~1 s. The computer used in this experiment was an ASUS Vivo laptop (ASUS, Hong Kong, China) with an Intel i5-9300h processor and 12 GB of memory.

### 3.1. Performance Evolution

The RUL calculation method is shown in Equation (14) [22], where $\bar{r}(t_{p})$ is the predicted value of the RUL, $N_{total}$ is the total number of predictions when the predicted value reaches the FT, and ΔT is the sampling period of the database. In this study, the IMS database sampling period was 10 min, and the FEMTO-ST database sampling period was 10 s.

(14) $$\bar{r}(t_{p})=N_{total}\cdot \Delta T$$

When the RUL was calculated for all of the data, the performance test of the prediction result was carried out. The first step was to construct the α-λ performance [27], which is a method to measure the performance of prediction and determine whether the prediction results are within an acceptable error range, where 𝛼 represents the error limit of the RUL prediction and λ is the given point and end of life (EOL) [27], converting the actual RUL to a 0–1 scale. For example, when λ = 0.6, it means that the bearing life has reached three-fifths of the EOL. When λ = 1, it means that the bearing has reached its EOL. The detection can be calculated as shown in Equation (15), where $r(t_{p})$ is the actual RUL, $\bar{r}(t_{p})$ is the predicted RUL, and the α error limit is set at 30%:

(15) $$\left[\left(1-\alpha )r(t_{p}\right)\right]\leq \;\bar{r}(t_{p})\leq \left[\left(1+\alpha )r(t_{p}\right)\right]$$

The second step is calculating the CRA. The relative accuracy (RA) of the measured value can be determined by subtracting the difference between the actual value and the measured value from the actual value, and then dividing by the actual value, as shown in Equation (16) [23]. The calculation of the average of the sum of multiple RA values is called the CRA, and the calculation is shown in Equation (17) [27]. In this study, the CRA obtained from data points with the same interval was selected to evaluate the accuracy of life expectancy.

(16) $$RA=1-\frac{\left|r(t_{p})-\bar{r}(t_{p})\right|}{r(t_{p})}$$

(17) $$CRA=\frac{1}{EOL-P+1}\overset{EOL}{\underset{i=P}{\sum }}RA$$

### 3.2. IMS Prediction Results

In the IMS experiments, the bearings Test 2 Bearing 1, Test 1 Bearing 4, and Test 3 Bearing 3 were selected, as they caused the main damage. Test 2 Bearing 1 was used as training data, while Test 1 Bearing 4 and Test 3 Bearing 3 were used as test data. The parameters obtained after training are shown in Table 3. The window boxes were all 60. In the ICMA, the norm value is less susceptible to the influence of the clutter wave, and the value is 7. In the gradient method, the gradient is more likely to fluctuate due to the influence of the clutter wave. In order to make the detection more accurate, we used a value of 20. In the use of the norm value, the M value of TSP is 1.125 times the $Norm_{mean}$, and $Norm_{TSP}$ is the $Norm_{mean}$ at the moment when the TSP is detected. When $Norm_{i}$ is greater than $1.4\times Norm_{TSP}$, it indicates the beginning of fast growth; when it exceeds 1.64 × $Norm_{TSP}$, it indicates that the FT will occur. In the gradient method, when the gradient exceeds 0.00055, it indicates the occurrence of the TSP; when the gradient is greater than 0.00077, it indicates the beginning of rapid destruction, and when it exceeds 0.00095 it indicates the occurrence of the FT.

**Figure 10:**
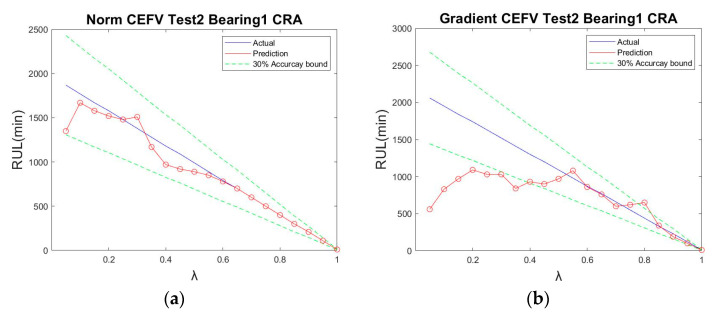
IMS Test 2 Bearing 1 CRA: (**a**) using the norm tracking value; (**b**) using the gradient tracking value.

Figure 10 shows the CRA diagram of IMS Test 2 Bearing1. We selected the data of 20 points to calculate the CRA. Through the calculation shown in Equation (17), using the result of the norm value, the CRA was determined to be 0.9450, which is equivalent to a 95% correct prediction rate. The result using the gradient value was 0.7459, corresponding to a 75% correct prediction rate.

**Figure 11:**
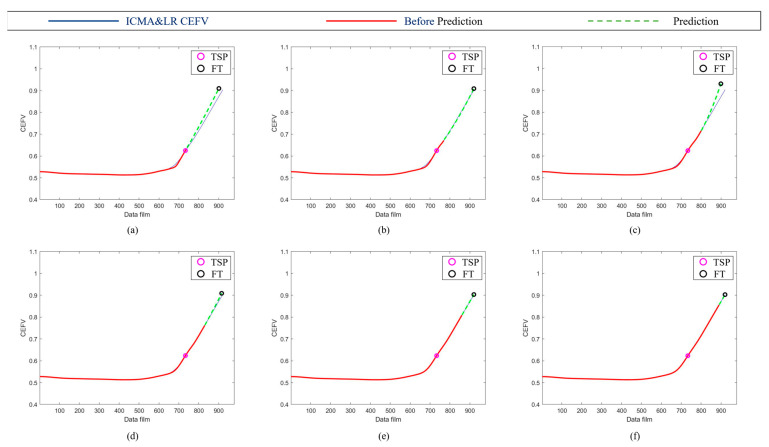
Trajectories of bearings’ health indicators using norm values. The trajectory was generated at different time points: (**a**) 7400, (**b**) 7700, (**c**) 8000, (**d**) 8300, (**e**) 8600, and (**f**) 8900 min.

**Figure 12:**
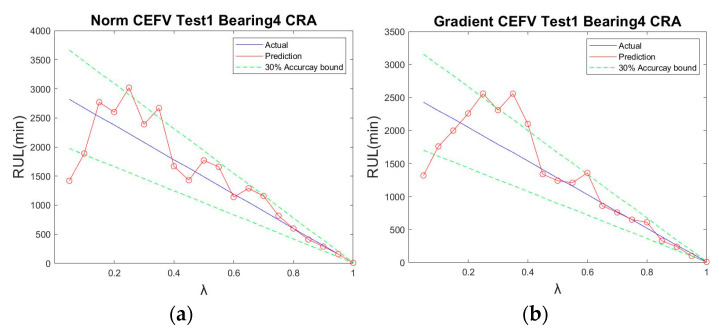
CRA of IMS Test 1 Bearing 4: (**a**) using the norm tracking value; (**b**) using the gradient tracking value.

**Figure 13:**
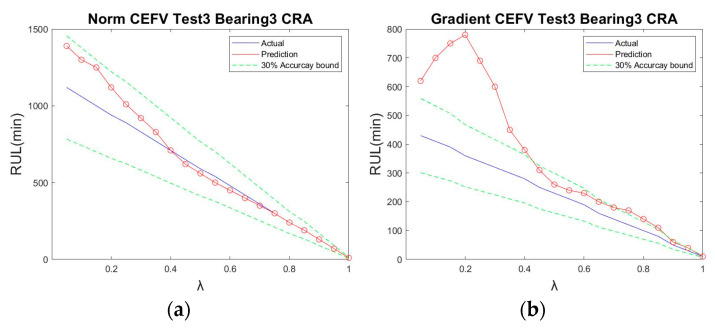
CRA of IMS Test 3 Bearing 3: (**a**) using the norm tracking value; (**b**) using the gradient tracking value.

Because the norm had better results, we further used the norm to plot the RUL prediction trajectory for different test points. The trajectory of the bearings’ HIs using the norm tracking value is shown in Figure 11. The trajectory was generated at different time points (7400, 7700, 8000, 8300, 8600, and 8900 min); the predicted results were very similar to the data after ICMA, demonstrating that the proposed method is effective for the assessment of the RUL of the ball bearings at different times. Moreover, the prediction results of Test 1 Bearing 4 and Test 3 Bearing 3 are shown in Figure 12 and Figure 13, respectively, to verify the performance of the proposed method.

Table 4 shows the CRAs of all of the data with the main damage, and the results for the training data are highlighted in orange. It can be seen that, when using the norm as the tracking value to predict the bearings’ RUL, the obtained prediction results are better than with the use of the gradient, and using the CEFV as the HI can obtain a better CRA.

### 3.3. FEMTO-ST Prediction Result

**Figure 14:**
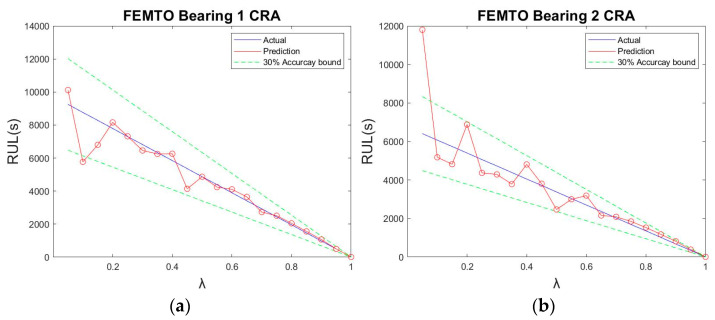

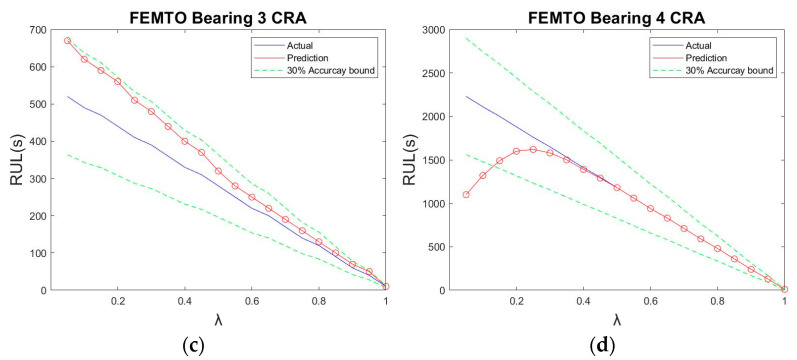
The CRA of the proposed method for the FEMTO-ST Test 1 database: (**a**) Bearing 1; (**b**) Bearing 2; (**c**) Bearing 3; and (**d**) Bearing 4.

**Figure 15:**
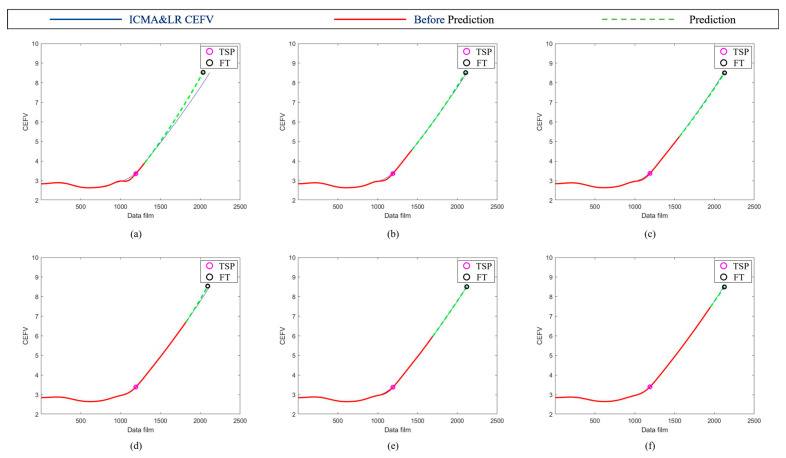
Trajectories of bearings’ health indicators using norm values. The trajectory was generated at different time points: (**a**) 13,000, (**b**) 14,300, (**c**) 15,600, (**d**) 16,900, (**e**) 18,200, and (**f**) 19,500 s.

The CEFV and norm method proposed in this paper was applied to the horizontal data of FEMTO-ST Test 1 for verification. After training, the experimental data parameters were as shown in Table 5. The performance of the proposed method after testing is shown in Figure 14, while Figure 15 shows the predicted trajectory of Bearing 1 using norm values.

Table 6 provides the CRA scores of the applied method and those used in [35] for comparison. As can be seen from Table 4, our method can achieve good prediction results.

### 3.4. Discussion

In the IMS data, the CEFV value can better represent the bearing degradation than the previous RMS. On the other hand, we used the norm and gradient to detect the RUL. Using the norm value provided a better performance in our method. Additionally, our proposed method was validated on FEMTO data. Although some data had not yet been predicted, the data collection was stopped because the vibration of the machine exceeded 20 g, resulting in a failure in the prediction. However, judging from the successful results, our proposed method still provided good prediction results.

## 4. Conclusions

This paper proposes a smart system combined with a regression prediction system to predict the RUL of ball bearings. Based on the results of the experiments described in this paper, the proposed method can achieve good results in the prediction of bearings’ RUL. Four conclusions can be drawn: (1) The HI after LPF and ICMA will show a smooth curve, which can improve the accuracy of prediction, and ICMA can make the curve smoother than the general SMA. (2) In this study, the Euclidean norm was used as the tracking value. The Euclidean norm is good for tracking the process of bearings’ degradation. Compared with the gradient in the RUL prediction, the effect of using the Euclidean norm was better than that of the gradient. (3) Through the method used in our experiments, the average CRA could exceed 90% in the prediction results of IMS data. (4) Applying the method proposed in this paper to the FEMTO-ST experimental data, the prediction also achieved good results. This study can be considered as a first step; thus, more extensive studies should be conducted in the future to further verify the effectiveness of the proposed method. In future research, deep learning tools should be taken into consideration for feature extraction, along with more appropriate TSP decisions and accuracy improvement, where a chaotic mapping strategy can also be applied to develop the main health indicators.

## Figures and Tables

**Table 1:** Three test conditions for the IMS dataset.

System Information	Conditions		
Test	1	2	3
Speed	2000 rpm		
Load	6000 lb		
Sampling rate	20 kHz		
Recording interval	1 s/every 10 min		
Channels	8	4	4
File	2156	984	6324

**Table 2:** Three test conditions for the FEMTO-ST dataset.

System Information	Conditions		
Test	1	2	3
Speed	1800 rpm	1650 rpm	1500 rpm
Load	4000 N	4200 N	5000 N
Sampling rate	25.6 kHz		
Recording interval	0.1 s/every 10 s		
Training data	Bearing 1_1 Bearing 1_2	Bearing 2_1 Bearing 2_2	Bearing 3_1 Bearing 3_2
Test data	Bearing 1_3 Bearing 1_4 Bearing 1_5 Bearing 1_6 Bearing 1_7	Bearing 2_3 Bearing 2_4 Bearing 2_5 Bearing 2_6 Bearing 2_7	Bearing 3_3

**Table 3:** IMS dataset prediction parameters.

Parameter	Conditions	
HI	CEFV	
Tracking values	Norm	Gradient
Window box	60	60
ICMA	7	20
TSP	$1.125\times Norm_{mean}$	0.00055
Fast growth	$1.400\times Norm_{TSP}$	0.00077
Failure threshold	$1.640\times Norm_{TSP}$	0.00095

**Table 4:** IMS data CEFV CRA comparison.

Parameter	Condition	
HI	CEFV	
Track value	Norm	Gradient
Test 2 Bearing 1 CRA	0.9450	0.7459
Test 1 Bearing 4 CRA	0.8355	0.8206
Test 3 Bearing 3 CRA	0.9230	0.5507
CRA AVG	0.9012	0.7057

**Table 5:** FEMTO-ST dataset prediction parameters.

Parameter	Conditions
HI	CEFV
Tracking values	Norm
Window box	60
ICMA	11
TSP	$1.15\times Norm_{mean}$
Fast growth	$2.00\times Norm_{TSP}$
Failure threshold	$2.95\times Norm_{TSP}$

**Table 6:** FEMTO-ST CRA comparison.

	Gebraeel Method	Linear Method	NWP Method	Our Method
Bearing 1	0.6230	0.5890	0.6960	0.9259
Bearing 2	0.5411	0.7771	0.8429	0.8361
Bearing 3	0.6961	0.6402	0.7808	0.8222
Bearing 4	0.6876	0.7573	0.7647	0.9276

## Data Availability

No new data were created in this paper.
